# Computational prediction of dust deposition on solar panels

**DOI:** 10.1007/s11356-022-22993-y

**Published:** 2022-09-16

**Authors:** M. Mekawy Dagher, Hamdy A. Kandil

**Affiliations:** 1 School of Engineering, Coventry University Branch in Egypt, The Knowledge Hub Universities, New Administrative Capital, Residential Area 7, R7, Cairo, Egypt; 2 Faculty of Engineering and Materials Science, The German University in Cairo, New Cairo, Cairo, 11385 Egypt

**Keywords:** Solar PV panels, CFD, Dust deposition, Cairo

## Abstract

This research is concerned with performing computational fluid dynamics (CFD) simulations to investigate the air flow and dust deposition behavior around a ground-mounted solar PV panel. The discrete phase model (DPM) is adopted to model the gas-solid flow. The influence of the wind speed, the dust particle size, and the dust material on the dust deposition rate was investigated based on the environment of Cairo, Egypt. The wind speeds range between 1 and 11.5 m/s with an average of 3.7 m/s. It is found that increasing the wind speed decreases the dust deposition rate. For wind speeds higher than 2 m/s, it is found that increasing the dust particle diameter or the dust density increases the dust deposition rate. For wind speeds lower than 2 m/s, it is found that there is a critical particle size before which increasing the dust density causes dust deposition rate to increase and after which increasing the dust density decreases the dust deposition. The maximum percentage of deposition rate equals 10.8% and occurs for the dolomite dust material at a wind speed of 2 m/s and particles diameter of 150 μm.

## Introduction

A severe problem facing the society today is to find sufficient energy for the future. Renewable energies are continuously renewed by nature. Solar energy is the largest and most abundant source of renewable energy that provides the earth with a huge amount of energy (Ellabban et al. 2014). Moreover, according to the renewable annual report by Murdock et al. (2019), solar PV panels have become the fastest growing energy technology in the world. Solar energy output is affected by two major parameters, which are temperature and dust deposition (Chiteka et al. 2019; Kalogirou et al. 2013). The accumulation of dust on the solar PV panel blocks the sunlight and degrades the solar transmittance, which in turn affects the solar PV power efficiency (Beattie et al. 2012; Zaihidee et al. 2016). Solar PV panels are strongly affected by soiling in regions with high concentration of airborne dust like the Middle East and North Africa (MENA) region (Ilse et al. 2016), while this problem is not significant in regions with heavy rain and/or snow with very low frequency of dust storms.

The influence of dust deposition on the solar PV panels is currently investigated either by experimental work or by numerical methods. Starting with the experimental research, Elminir et al. (2006) examined the impact of dust deposition on the power performance of the solar PV panels. The study indicated that the dust accumulation increases as the tilt angle is decreased. Moreover, it was found that, for a panel installed with a tilt angle of 45° and facing south, the decrease in the output power was about 17.4% per month. In the Eastern province of Saudi Arabia, Adinoyi and Said (2013) investigated the effect of dust deposition on the output power of solar PV panels. Their results indicated that the output power could decrease by more than 50% if the panels were not cleaned for a period of more than 6 months. The study also found that dust sticks on the glass of the panels due to humidity, so it requires strong but careful cleaning to restore the initial performance. Essalaimeh et al. (2013) analyzed a system of solar energy in Amman, Jordan, and the experiments revealed that dusty panels produced 31–35% less power than clean panels on the maximum solar intensity. In the Kingdom of Bahrain, Alnaser et al. (2015) investigated the effect of dust deposition on the power generated by the 0.5 MW solar PV system. It was found that the dust deposition density ranged between 5 and 12 g/m^2^. The power was recorded to drop to an average of 40% of the maximum available power. The effect of soiling on the output power of solar PV panels with 22° tilt angle was investigated in Qatar (Ilse et al. 2016). It was found that a solar PV array cleaned every 2 months generated 8.1% less energy than an identical array cleaned weekly. Moreover, never cleaning the panels produced 22.8% less energy. Anana et al. (2016) studied the influence of soiling on high concentration PV module performance in Morocco. Their results indicated that the module performance dropped by 7.64% for a 1.61-μm-thick fouling layer in 1 month. In The United Arab of Emirates, Hachicha et al. (2019) analyzed the dust deposition and its influence on the electrical characteristics of the solar PV modules. The study found that high dust densities reduced the short circuit current greatly. A linear relationship was also found between the normalized solar PV power and the dust deposition with a drop of 1.7% per each g/m^2^ of dust. In Europe, Ghazi and Ip (2014) studied the effect of dust and other solid particle deposition on the performance of the solar PV panels in Brighton, the United Kingdom (UK). Their results from outdoor experiments showed only 5% reduction of transmittance after 4 weeks. This means that dust deposition in rainy winter in the UK is low. In Spain, Piliougine et al. (2013) studied the effect of adding self-cleaning coating to the solar PV panels. It was found that the modules with coating film had a yearly average daily energy soiling losses of 2.5%, while the uncoated modules had energy losses of 3.3%. In California, USA, Mejia and Kleissl (2013) found that the influence of soiling on PV panel performance is low and hardly guaranteed the cleaning expenses of the solar PV panels. In South America, Fraga et al. (2018) investigated the effect of solar PV panel soiling on the performance of solar PV power plant in the state of Minas Gerais, Brazil. The results showed that soiling decreased the peak power by 13.7% in the dry period and 6.5% in the period after rainfall. In China, Jiang et al. (2011) found that the reduction in the PV output efficiency increased from 0 to 26% when dust deposition density increased from 0 to 22 g/m^2^. Chen et al. (2020) investigated the influence of soiling on the solar PV power output in East China. It was found that the dust accumulation reached 0.644 g/m^2^ after 1 week of exposure in the dry season, which resulted in a power loss of 7.4%.

Few studies used the numerical methods of computational fluid dynamics (CFD) to investigate the dust behavior around the solar PV panels, although they have become a powerful tool in modeling the fluid motion including dust deposition (Wu et al. 2020). For example, Lu et al. (2016) investigated the dust deposition characteristics on solar PV panels. The results showed that the dust deposition rate increased reaching a peak value and then decreased with the increase of dust particle diameter. The maximum deposition rate was 0.28% for dust particle size of 10 μm, and minimum deposition rate was 0.13% for dust particle size of 50 μm. Lu and Zhao (2018) analyzed the influence of dust particle diameters and tilt angles of the solar PV panel on the dust deposition behaviors. The results showed that the maximum deposition rates were 14.28%, 13.53%, 6.79%, and 9.78% for solar PV tilt angles of 25^°^, 40^°^, 140^°^, and 155^°^, respectively. Lu and Zhao (2019) investigated the dust deposition rate on a PV panel for different particle diameters and wind speeds. For the 100-μm-diameter particles, the peak deposition rate was 13.71% at a wind speed of 1.3 m/s. However, the peak deposition rate was 14.28% for the 150-μm-diameter particles at a wind speed of 2.6 m/s. Chiteka et al. (2019) investigated the influence of the installation parameters on the dust deposition for ground mounted solar PV panels. Wu et al. (2020) predicted the dust deposition for different wind velocities and wind directions based on the environment of Liverpool. The results indicated that the solar PV output power decreased by a ratio from 46 to 70% due to dust deposition and temperature increase. Dagher and Kandil (2022) investigated the installation of vortex generators as a novel passive cleaning method of the solar PV panels. The results showed that a dust deposition reduction of 35% can be achieved.

Reviewing the literature showed that few articles employed the numerical methods to examine the dust characteristics in the flow around the solar PV panels. Egypt is one of the sunniest places in the world; however, it strongly suffers from the problem of dust accumulation because of the nature of its environment. The dust deposition behaviors based on Cairo, Egypt, environmental properties have not been numerically studied yet. Moreover, the influence of different dust materials on the dust deposition rate has not been numerically investigated. Understanding the dust deposition behavior is very important in finding possible solution strategies to reduce the deposition. For example, cleaning methods can be designed to be more effective at high dust deposition rate situations. Consequently, the results of this research are used to investigate a passive cleaning method by Dagher and Kandil (2022).

In this regard, the objective of this study is to investigate the dust deposition behaviors based on Cairo, Egypt’s environmental properties. It is aimed to examine the impact of the wind speed, the dust particle size, and the dust material on the dust deposition rate on the PV panels.

## Numerical methodology

This study is concerned with the numerical investigation of the air flow and dust behavior around the solar PV panels, using a two-dimensional (2D) computational fluid dynamics (CFD) simulation. Modeling 2D air and dust flow around the solar PV panel is an accepted approximation, since the panel cross section does not change in the third dimension. ANSYS Fluent 18.2 software is used to perform the simulations. Reynolds-Averaged Navier-Stokes (RANS) equations are solved by the software. The shear stress transport (SST) *k* – *ω* turbulence model, developed by Menter (1994), is used as a closure because it was found that it behaves well in modeling turbulence in the air flow around solar PV panels and in dust deposition applications (Chiteka et al. 2019; Jubayer and Hangan 2014; Lu and Zhao 2019). The discrete phase model (DPM) is adopted to predict the dust deposition behavior.

### Air flow fields

Turbulent fluid flow is governed by the RANS equations that are based on the laws of the conservation of mass and momentum. For incompressible flow, the RANS equations are written as follows:

1 $${~}^{\partial {U}_i}\!\left/ \!{~}_{\partial {x}_i}\right.=0$$

2 $$\rho {~}^{\partial {U}_i}\!\left/ \!{~}_{\partial t}\right.+\rho {U}_j\ {~}^{\partial {U}_i}\!\left/ \!{~}_{\partial {x}_j}\right.=-{~}^{\partial P}\!\left/ \!{~}_{\partial {x}_i}\right.+{~}^{\partial }\!\left/ \!{~}_{\partial {x}_j}\right.\left(2\mu {S}_{ji}-\uprho \overline{u_j^{\prime }{u}_i^{\prime }}\right)$$

Equation (1) is the conservation of mass, and the conservation of momentum is represented by Eq. (2), where *ρ* is the fluid density; *μ* is the dynamic viscosity of the fluid; and *P* is the static pressure. *U*_*i*_ are the time-averaged fluid velocity components. The term 2*μS*_*ji*_ is the viscous stress tensor where *S*_*ji*_ is represented by Eq. (3). The last term on the right-hand side of Eq. (2) represents the force due to turbulence per unit volume where $\uprho \overline{u_j^{\prime }{u}_i^{\prime }}$ is the Reynolds stress tensor and ${u}_i^{\prime }$ represents the fluctuation velocity of the fluid. Reynolds stresses make the number of unknowns higher than the number of RANS equations. Consequently, a turbulence model should be used in order to close the equations (Wilcox 2006). The shear stress transport (SST) *k* – *ω* turbulence model is adopted as a closure to predict the turbulent air flow around the solar PV panel. The solution is considered converged when a residual value of 10^−6^ is obtained.

3 $${S}_{ji}=\frac{1}{2}\left({~}^{\partial {U}_i}\!\left/ \!{~}_{\partial {x}_j}\right.+{~}^{\partial {U}_j}\!\left/ \!{~}_{\partial {x}_i}\right.\right)$$

### Dust motion and behavior modeling

The flow in this study is a gas-solid flow which is a multiphase flow. The gas phase is represented by the air, and the solid phase is represented by the dust particles. There are two approaches for the numerical modeling of multiphase flows which are the Euler-Lagrange approach and the Euler-Euler approach. The Euler-Lagrange approach is suitable when the discrete phase occupies a low fraction volume that can be neglected (ANSYS 2013). Consequently, the Euler-Lagrange approach is appropriate for modeling the dust deposition around the solar PV panels (Chiteka et al. 2019). Since the dust occupies a low volume fraction of the air, the interaction between dust particles and their impact on the air flow are neglected in the current study. The discrete phase model (DPM) in Ansys Fluent software follows the Euler-Lagrange approach, so it is utilized in this study. The equation of motion of the discrete phase particles is the momentum equation as described by Eq. (4).

4 $${m}_p{~}^{d\overrightarrow{u_p}}\!\left/ \!{~}_{ dt}\right.=\left(\frac{1}{2}{C}_D{A}_p\ \rho \left|\overrightarrow{u}-\overrightarrow{u_p}\right|\left(\overrightarrow{u}-\overrightarrow{u_p}\right)\right)+{m}_p\overrightarrow{g}-\rho {V}_p\overrightarrow{g}+\overrightarrow{\mathrm{F}}$$

The right-hand side terms of Eq. (4) represent the different forces acting on a single dust particle. The first term represents the drag force; the second term, ${m}_p\overrightarrow{g}$, represents the weight of the particle; and the third term; $\rho {V}_p\overrightarrow{g}$, represents the buoyancy force. Moreover, *m*_*p*_, *u*_*p*_, *A*_*p*_, and *V*_*p*_ are the particle mass, velocity, cross-sectional area, and volume, respectively. The drag coefficient is represented by *C*_*D*_. The last term of the right-hand side of Eq. (4); F represents other additional forces that may be added under special circumstances (ANSYS 2013). The Saffman’s lift force which is the lift due to shear, described by Li and Ahmadi (1992), is the additional force included in the current model. The virtual mass and pressure gradient forces are ignored in this study because the particle density, *ρ*_*p*_, is much higher than the air density, *ρ*. The Brownian force is also neglected because it is intended for sub-micron particles and for laminar simulations (ANSYS 2013). The discrete random walk model (DRW) is activated to predict the particles dispersion due to turbulence.

### Computational domain

**Fig. 1:**
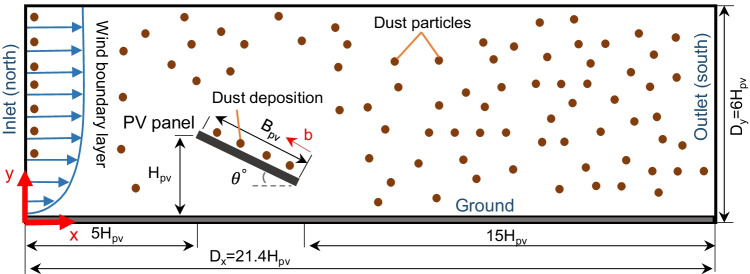
Schematic of the domain used for the CFD simulations

The geometry of the computational domain is created using Solidworks 2019 software. Fig 1 shows a schematic of the domain dimensions used for the simulations. The PV system geometry is designed to be in consistence with the wind tunnel experiments of Abiola-Ogedengbe et al. (2015) for the sake of validation. The dimensions of the computational domain are selected to be the same as the domain of Jubayer and Hangan (2014), following the cost guidelines of Franke et al. (2007). The height of the PV panel from the ground, *H*_pv_, equals 1.65 m. The breadth of the PV panel, *B*_pv_, equals 2.48 m. The tilt angle *θ* equals 25^°^. The length of the domain in the *x* direction, *D*_*x*_, equals 21.4 *H*_pv_. The height of the domain in the *y* direction: *D*_y_, equals 6 *H*_pv_. The distance from the leading edge along the breadth of the panel is b.

**Fig. 2:**
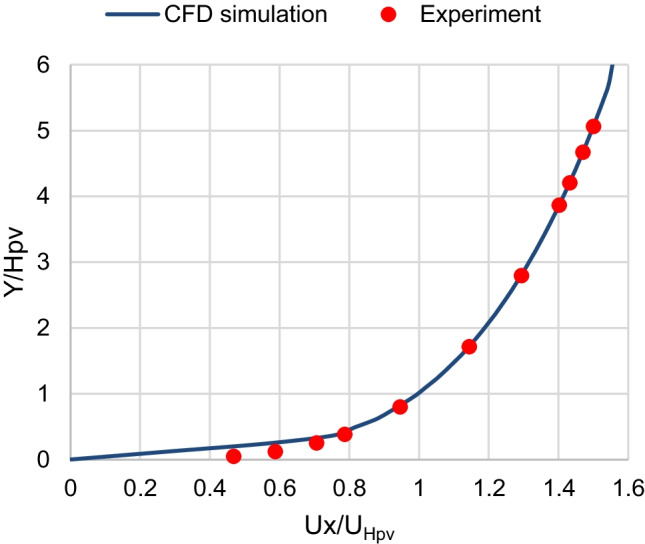
Inlet wind velocity profile

**Fig. 3:**
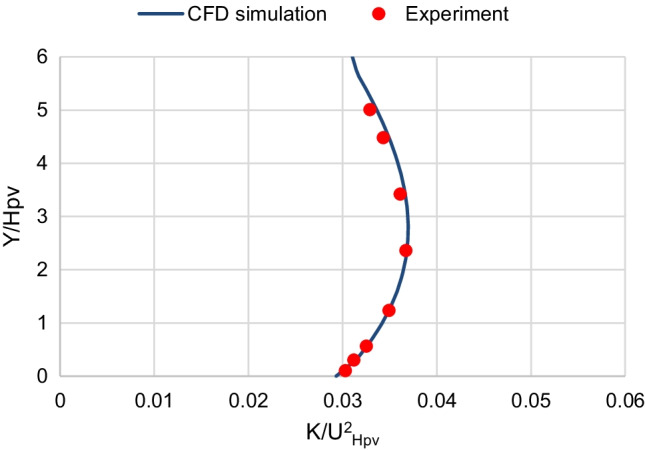
Inlet turbulent kinetic energy (TKE) profile

For the boundary conditions, the inlet wind velocity and turbulent kinetic energy (TKE) profiles are set as user-defined functions (UDF) to match the profiles of Tominaga’s (2015) experimental data that corresponds to the wind boundary layer of a suburban terrain. Consequently, this study simulates the wind boundary layer of the suburban regions in Cairo. The inlet velocity profile is fitted to a power function, and the inlet TKE profile is fitted to a polynomial function. Figs. 2 and 3 show that the wind velocity and the TKE profiles at inlet agree well with that of the experimental measurements, respectively.

Where *U*_*x*_ is the *x* component of the wind velocity at height *y* from the ground, *U*_Hpv_ is the wind velocity at height *H*_pv_; and *K* is the turbulent kinetic energy. The no-slip boundary condition is applied at the walls of the ground and the panel. The symmetry boundary condition is applied at the upper boundary of the domain. The standard atmospheric pressure of zero pressure gauge is applied at the outlet, where the pressure outlet boundary condition is used.

To simulate the dust flow, 100,000 dust particles are injected from the inlet boundary. The injected number of dust particles is based on the available computational resources. The trap boundary condition for the discrete phase is applied to the upper surface of the PV panel. Therefore, the particles rebound at walls are not considered in the current study. The escape boundary condition is applied at the other boundaries of the domain where the dust particles are assumed to leave the computational domain and are lost from the calculation when they get in contact with these boundaries. The percentage of dust deposition rate is used to compare between different simulation cases. The percentage of dust deposition rate is defined as the number of trapped dust particles over the total number of injected particles in order to make the comparison independent of the injected number of dust particles.

### Grid independence test

**Fig. 4:**
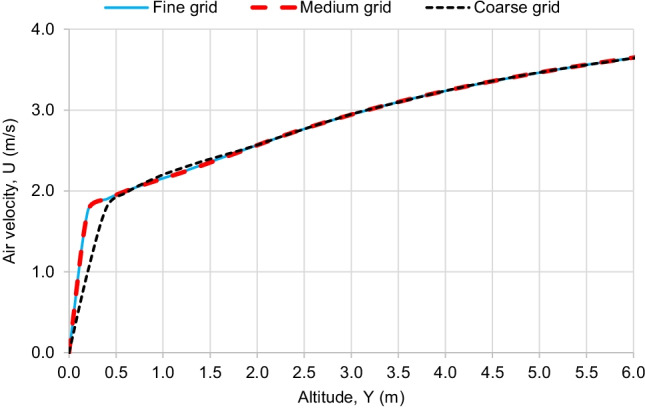
Velocity profiles at distance *X* = 7 m from inlet for different grids

To determine the minimum number of mesh cells that can be used in the simulations without affecting the results, a grid independence test is performed. Four grids were created, including a coarse mesh with 24,034 cells, a medium mesh with 55,257 cells, a fine mesh with 79,273 cells, and a very fine mesh with 102,826 cells. The air flow velocity profiles, at a vertical line of 7-m distance from the inlet, are compared for the different grids. Fig 4 shows the comparison between the velocity profiles of the different grids. It is found that the results of coarse mesh are slightly different than those of the other grids. However, the medium, fine, and very fine meshes have almost the same velocity profile. At lower altitude, there is a high change in the air velocity because of the wind boundary layer, so the coarse grid could not predict that change precisely. However, at higher altitude, the velocity value becomes nearly constant, and that is why, all the different grids were able to predict the air velocity. Therefore, the fine mesh is chosen for further simulations. The very fine grid velocity profile is excluded from Fig. 4 to simplify the graph, as it has the same result of the medium and fine grids.

## Results and discussions

The important parameters of wind and dust particles are selected based on the environmental data of Cairo, Egypt, as shown in Table 1. Wind velocity values are chosen based on the research of Essa and Mubarak (2006), where the wind speed in Cairo ranges throughout the year between 1 m/s and 11.5 m/s at 1-m height from the ground surface. Moreover, the average wind speed in Cairo is 3.7 m/s. As a result, the user-defined power function of the velocity profile at the domain inlet is entered such that the wind speed has the selected values in Table 1 at a height 10 m from the ground. Dust diameters and materials are assumed based on the research of Shaltout et al. (2016). The most frequently observed chemical compounds of dust in Cairo are dolomite of density, *ρ* = 2872 kg/m^3^, calcite of density, *ρ* = 2800 kg/m^3^, quartz of density, *ρ* = 2650 kg/m^3^, and gypsum of density, *ρ* = 2320 kg/m^3^. Hussein et al. (2004) found that the maximum yearly output energy in Cairo can be obtained from PV modules oriented facing south with tilt angles in the range between 20^°^ and 30^°^, and that is why, the selected tilt angle is 25^°^. Wind direction is north, since it is the most prevailing wind direction in Cairo throughout the year (Zakey et al. 2008).

**Table 1:** Environmental parameters of Cairo, Egypt used in the present CFD simulations

Environment	Wind speeds (m/s)	Dust particle diameters (μm)	Dust materials	Tilt angle
Cairo, Egypt	2, 4, 6, 9, and 11.5 (north)	1, 10, 30, 40, 50, 100, and 200	Dolomite, calcite, quartz, and gypsum	25^°^ (facing south)

### Model validation and verification

**Fig. 5:**
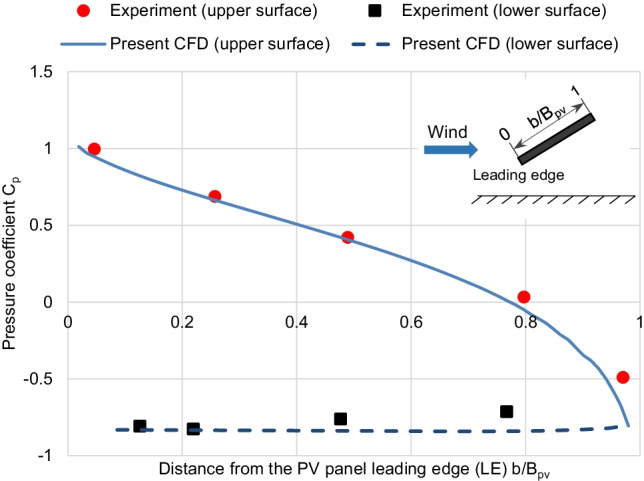
Validation of the pressure coefficient profile along the PV panel surface

Air flow fields are validated using the wind tunnel experimental measurements of Abiola-Ogedengbe et al. (2015). The pressure coefficient, *C*_p_, profiles are calculated on both the upper and lower surfaces of the panel from the results of the CFD simulations to be compared with the experimental measurements in the wind tunnel. Fig. 5 shows that the *C*_p_ profile of the current CFD simulation agrees with the measured values of the wind tunnel experiment by Abiola-Ogedengbe et al. (2015). Consequently, it is concluded that the present CFD model can accurately predict the air flow fields around the PV panel.

**Fig. 6:**
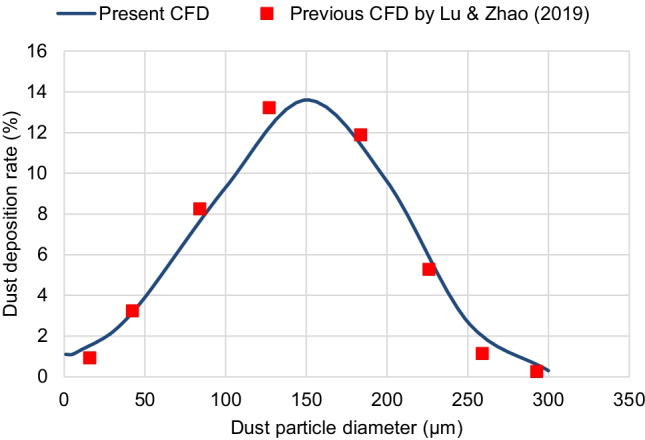
Verification of the dust deposition behavior of the present CFD simulations

The discrete phase model (DPM) is adopted to predict the dust deposition behavior around the solar PV panel. For the sake of verification, the present simulation results are compared with those of a previous CFD model by Lu and Zhao (2019). The same computational domain was created, and the same conditions were applied. The percentage of the dust deposition rate is calculated for dust particles of sizes between 1 μm and 300 μm. Fig. 6 shows that the dust deposition behavior predicted by the present CFD simulation agrees well with that of the previous CFD simulation by Lu and Zhao (2019). In consequence, it is decided that the present CFD simulations can accurately predict the dust deposition behavior.

### Air flow fields

**Fig. 7:**
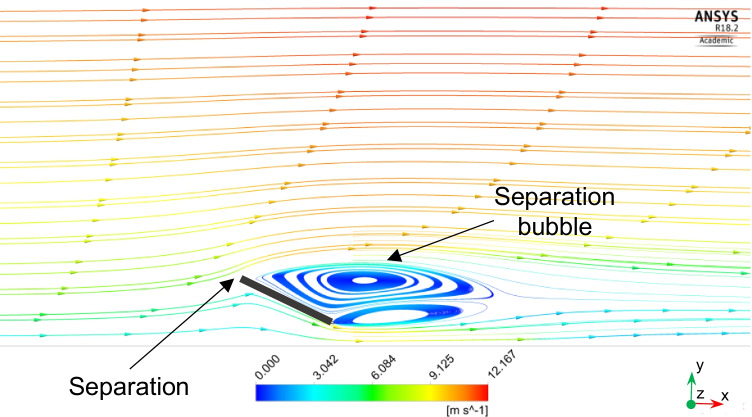
Air streamlines around the PV panel

**Fig. 8:**
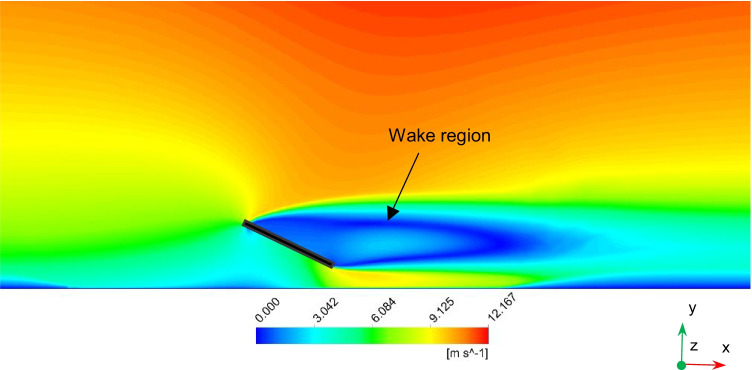
Velocity contours around the PV panel

**Fig. 9:**
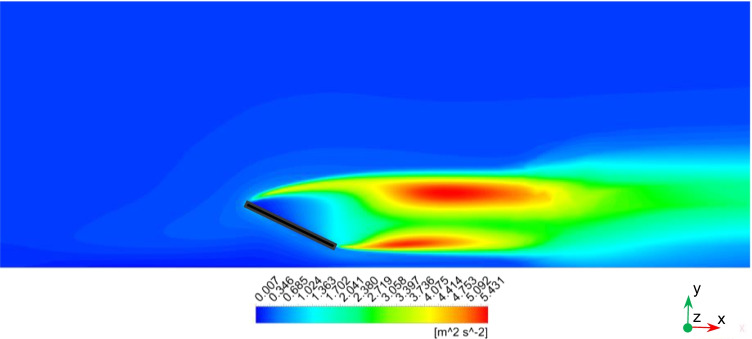
TKE distribution around the PV panel

Air flow fields for different wind velocities are analyzed because they are important for dust motion and deposition behavior. Streamlines, velocity distribution, and turbulence kinetic energy (TKE) distribution of the air flow around the solar PV panel are simulated and analyzed. Similar behaviors of the air flow fields are found for different wind speeds. As a result, the results are just shown for the highest wind speed of 11.5 m/s. Fig. 7 shows the air streamlines around the PV panel for wind speed of 11.5 m/s. Air flows from north to south, i.e., from the inlet to the outlet of the computational domain. When the streamlines approach the PV panel, they split and some of them move above the panel, while the others move beneath the panel. One of the properties of the streamlines is that they cannot move in sharp corners. Consequently, when the streamlines approach the PV panel, they cannot move on the panel surface and separation occurs at the upper edge of the PV panel. Flow separations are noticed at the upper and lower edges of the panel, and two separation bubbles are formed in front of the panel surface. Small dust particles are expected to follow the streamlines and deposit at lower rates on the panel surface because of their low weight. Large dust particles are more affected by gravitational forces, so they will have more deposition rates compared to small ones. However, by increasing wind velocity, aerodynamic drag force will have more effect on the particles than the gravitational force. In consequence, for high wind velocities, dust deposition will decrease for large dust particles. Fig. 8 shows the air velocity contours around the PV panel for wind speed of 11.5 m/s. It can be seen that velocity magnitude increases as the altitude increases because of the wind boundary layer. Moreover, due to flow separation, a wake region of low velocity values is formed in front of the panel. Fig. 9 shows the TKE distribution of the air around the solar PV panel. It can be seen that TKE values become high when the air flows few meters past the PV panel because of the air vortices formed in this region.

### Dust deposition

All combinations between the parameters in Table 1 are taken into consideration, and a total of 140 simulation cases are performed to predict the deposition for different dust particle diameters, different dust materials, and different wind speeds in Cairo. In this paper, the significant results of the simulations are highlighted.

**Fig. 10:**
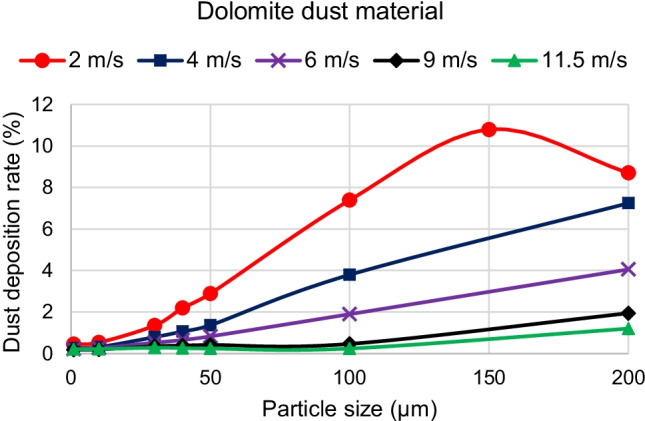
Effect of changing dolomite dust particle diameter on the dust deposition rate for different wind speeds of 2, 4, 6, 9, and 11.5 m/s

The first parameter is the effect of dust particle diameters. Fig. 10 shows the effect of changing dolomite particle diameter on the dust deposition rate for different wind velocities. It can be seen from the results of Fig. 10 that dust deposition rates are significantly different for varied dust particle sizes. For the studied range of particle sizes of Cairo, it can be concluded that dust deposition rate increases by increasing the dust particle diameter for wind velocities above 2 m/s. However, for wind velocity of 2 m/s, it is observed that dust deposition rate reaches its peak value at particle diameter of 150 μm, then the deposition rate starts to decrease. Simulations are performed for calcite, quartz, and gypsum, and it is found that they have similar behaviors.

The maximum percentage of deposition rate equals 10.8%, and it occurs for the dolomite dust material at a wind speed of 2 m/s and particle diameter of 150 μm. The minimum percentage of deposition rate occurs at the high wind speeds of 9 and 11.5 m/s and particle diameter of 1 μm, where all dust materials have nearly the same percentage of dust deposition rate of about 0.2%.

**Fig. 11:**
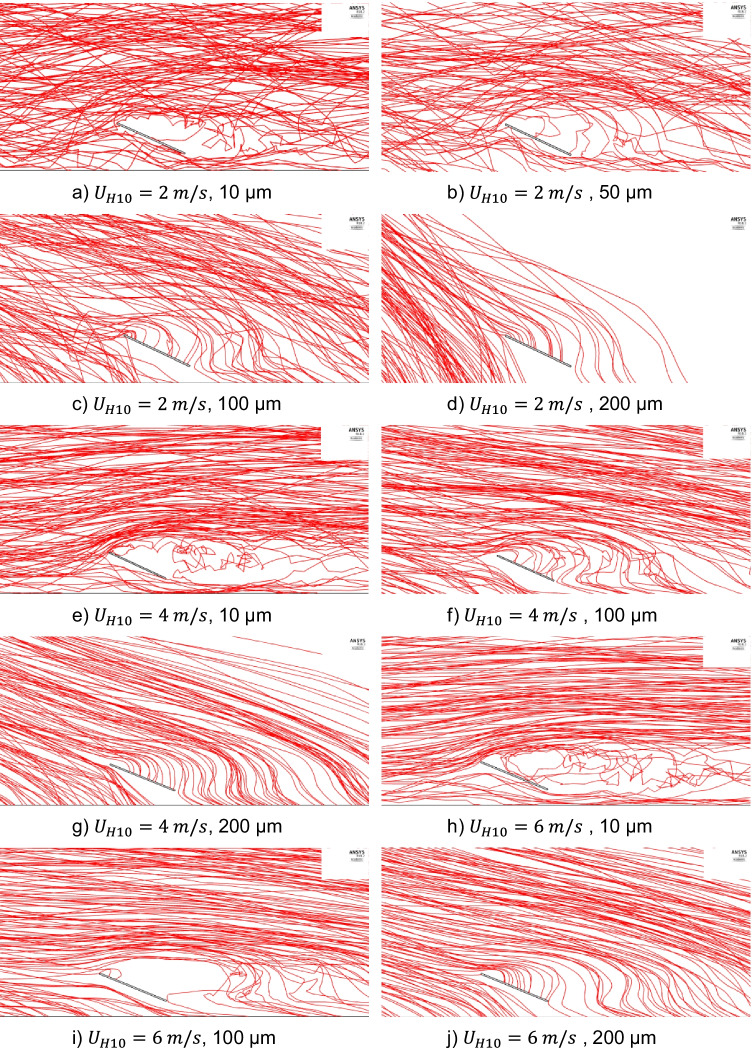
Dolomite dust particles trajectories for different particle sizes and wind velocities, *U*_*H*10_, of 2, 4, and 6 m/s

**Fig. 12:**
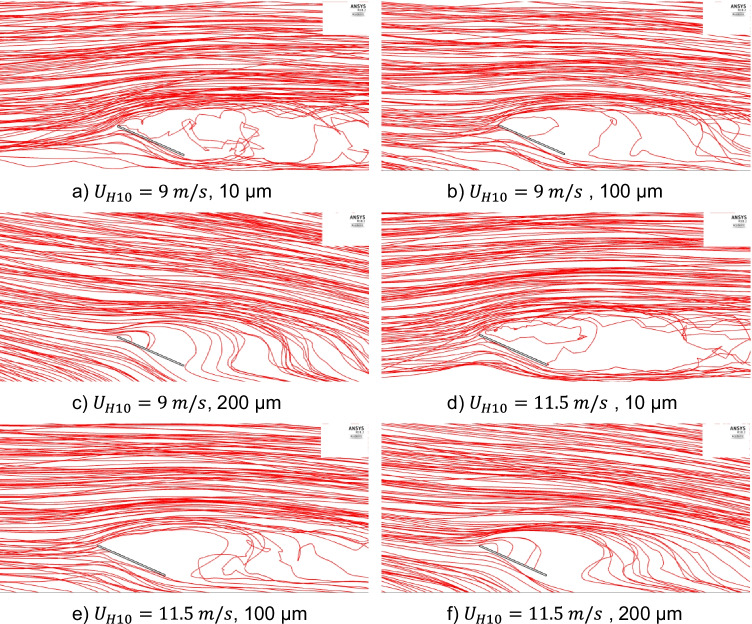
Dolomite dust particles trajectories for different particle sizes and wind velocities, *U*_*H*10_, of 9 and 11.5 m/s

Fig. 11 (a) to (j) show the dolomite dust particles’ trajectories of different particle sizes and wind velocities of 2, 4, and 6 m/s. Fig. 12 (a) to (f) show the dolomite dust particles’ trajectories of different particle sizes and wind velocities of 9 and 11.5 m/s. Only 200 trajectories are shown out of the 100,000 injected particles in order to be able to track and visualize them. Dust trajectories can explain the deposition behavior of Fig. 10.

For the wind velocity of 4 m/s, it can be observed from the dust trajectories in Fig. 11 (e) that for small particle sizes of 10 μm, the dust particles are carried by the air flow and follow the streamlines because of their low weight. Consequently, they have a low deposition rate. It can be also observed from Fig. 11 (f) and (g) that increasing the dust size to 100 μm and 200 μm, respectively, increases the gravitational effect and makes more particles deposit on the panel surface. Same observations can be noticed for wind velocity of 6 m/s in Fig. 11 (h) to (j) and wind velocities of 9 and 11.5 m/s in Fig. 12 (a) to (f). These observations explain the behavior of changing the dust particle size for the wind velocities of 4, 6, 9, and 11.5 m/s in Fig. 10.

For the wind velocity of 2 m/s, it can be observed from Fig. 11 (a), (b), (c), and (d) that increasing the particle size increases the gravitational effect and makes more particles tend to fall down than moving forward with the air flow. Consequently, more particles accumulate on the PV panel by increasing the dust particle diameter from 10 to 100 μm. However, Fig. 11 (d) shows that at particle size of 200 μm, most of the dust particles fall on the ground before reaching the PV panel, and that is why, the dust deposition rate decreases after reaching its peak. These observations explain the behavior of changing the dust particle size for the wind velocity of 2 m/s in Fig. 10.

**Fig. 13:**
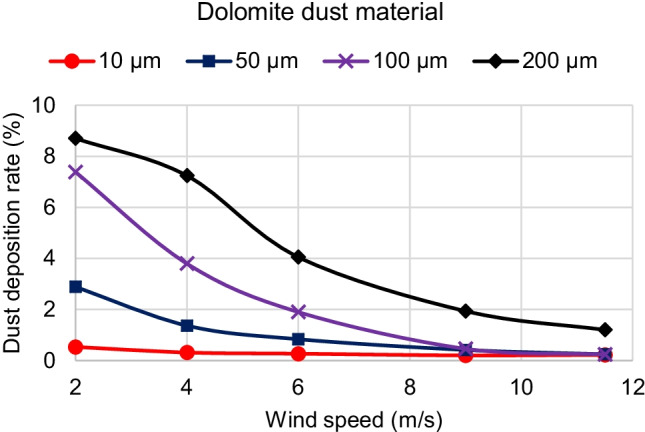
Effect of increasing the wind speed on dolomite dust deposition rate for different dust particle diameters

For the second parameter, which is the effect of wind velocity, Fig. 13 shows the effect of increasing the wind velocity on the dolomite dust deposition rate. It is observed that increasing the wind velocity decreases the dust deposition rate. This is because increasing the velocity increases the drag force which drives the dust particles to move with the air flow. Moreover, it is observed that by increasing the wind velocity, the difference in dust deposition rates between different particle sizes becomes smaller. In other words, for high wind velocities, above 8 m/s, the dust deposition rates of different particle sizes are nearly the same.

Dust trajectories also explain the behavior of dust deposition rate due to the wind velocity change. Fig. 11 (c), (f), and (i) and Fig. 12 (b), and (e) show the dolomite dust trajectories of the same particles’ diameter of 100 μm for different wind velocities of 2, 4, 6, 9, and 11.5 m/s, respectively. It is noted that as the wind velocity increases, dolomite of 100-μm diameter dust particles become more airborne and deposit less on the panel surface. Furthermore, Fig. 12 shows the dust trajectories for high wind speeds of 9 and 11.5 m/s. It is observed, for these high wind speeds, that dust trajectories are nearly the same for small and large size particles. Both small and large sized particles tend to move forward with the air flow than to fall down and accumulate on the panel. This is why, for high wind speeds above 8 m/s, the dust deposition rates of different particle sizes are low, and they have nearly the same value.

Changing the particle size changes its weight which in turn affects the accumulation rate. However, changing the density will have a different effect on the accumulation rate than changing the particle size. This is because the gravitational force is not the only force affecting the dust particles. There is also the aerodynamic drag force, and this force depends on the geometry and size of the particle. In other words, changing the dust density affects the gravitational force only but changing the dust particle size affects both the gravitational force and the drag force. Therefore, different dust particle sizes and different dust densities have different effects on the dust accumulation rate. Moreover, the same mass of two particles with different sizes and densities will lead to different dust accumulation rates. Consequently, the effect of changing the dust material on the accumulation rate has to be studied and investigated.

**Fig. 14:**
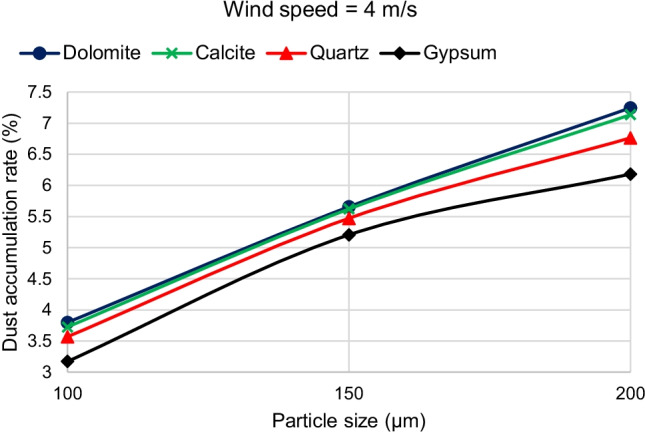
Effect of increasing dust density on dust deposition rate for different dust sizes at a wind velocity of 4 m/s

**Fig. 15:**
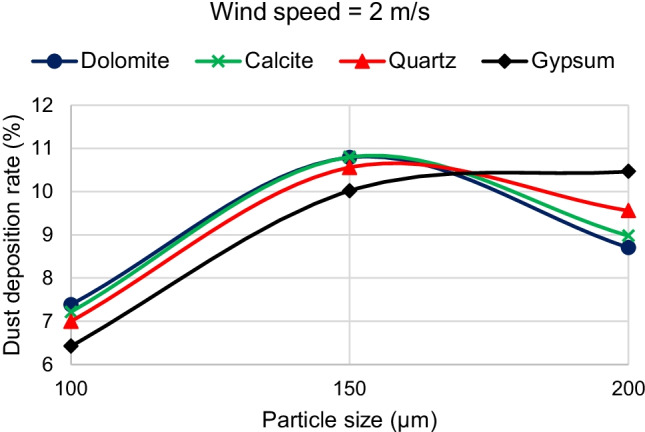
Effect of increasing dust density on dust deposition rate for different dust sizes at a wind velocity of 2 m/s

The third parameter is the effect of dust material. For Cairo, Egypt environment, dolomite has the highest density then calcite, quartz, and gypsum in a descending order. On the one hand, it is found that for wind velocities higher than 2 m/s, increasing the material density causes the dust deposition rate to increase for all dust sizes, as shown in Fig. 14. Figure 14 shows that at a wind velocity of 4 m/s, the material with higher density has a higher deposition rate. That is why, dolomite has the highest deposition rate, and gypsum has the lowest deposition rate for all dust particle sizes. This is because increasing the density increases the weight and makes more particles accumulate on the panel surface. On the other hand, it is found that, for wind velocities less than or equal to 2 m/s, dust deposition rate has a different behavior. It is observed that there is a critical particle size before which increasing density causes dust deposition rate to increase. After the critical particle size, increasing the density causes the dust deposition rate to decrease. This is because reviewing the dust trajectories show that in this region, increasing the dust density from gypsum to dolomite makes more dust particles fall on the ground before reaching the PV panel. Figure 15 shows that for a wind velocity of 2 m/s, increasing the dust density increases the dust deposition rate at 100-μm particle size. Dolomite has the highest deposition rate at 100-μm particle size. However, increasing the dust density decreases the dust deposition rate at a particle size of 200 μm. Dolomite has the lowest deposition rate at 200-μm particle size.

**Fig. 16:**
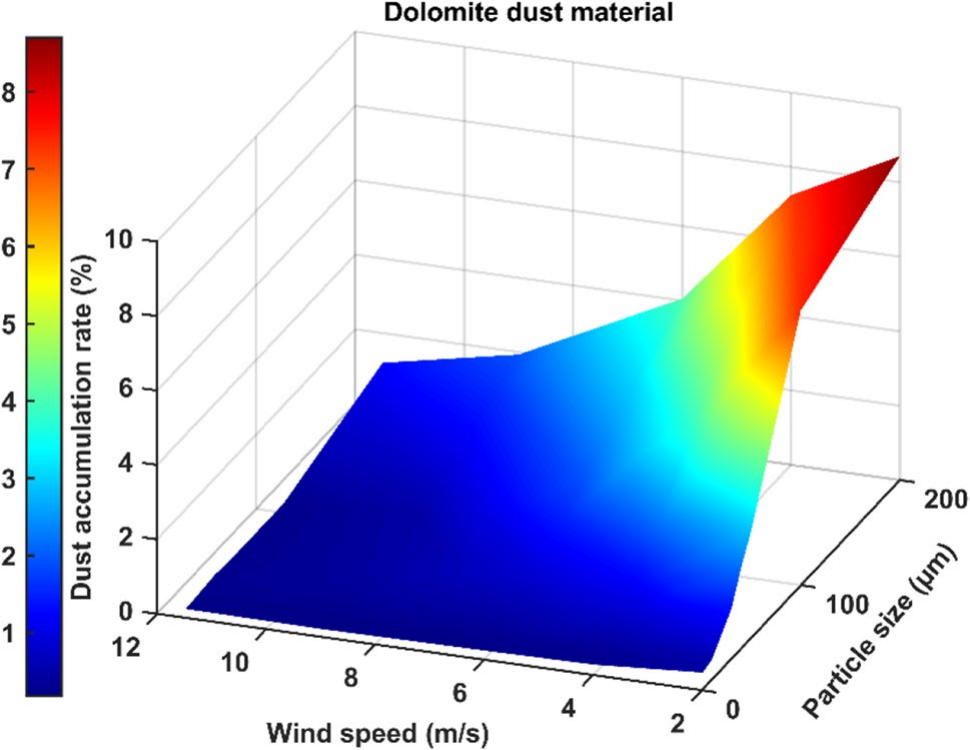
Effects of both dust particle size and wind velocity on the deposition rate for dolomite dust material

To summarize the results, three-dimensional graphs are created to better visualize the relation between different parameters. Figure 16 shows the effects of both dust particle size and wind velocity on the deposition rate for dolomite dust material. It can be seen from Fig. 16 that, for dolomite dust material, increasing the particle size or diameter increases the deposition rate for the majority of Cairo wind speeds. However, increasing the wind speed decreases the deposition rate. Similar behaviors are found for the other dust materials of calcite, quartz, and gypsum.

## Conclusion

Numerical simulations, using the computational fluid dynamics (CFD) techniques have become a powerful tool for predicting multiphase flow and solar energy applications (Gao et al. 2012; Lu and Zhao 2019). Moreover, few studies used these numerical methods to study the dust characteristics in the flow around the solar PV panels. Most of the studies focused on the performance of the PV panel after the dust accumulation. However, the behavior and characteristics of the dust deposition process itself have seldom been studied (Lu and Zhao 2019; Wu et al. 2020). Studying the effect of different dust materials on the dust deposition rate was not found in the literature. The important parameters of wind and dust particles are selected based on the data of Cairo, Egypt which has not been numerically investigated before.

The present study adopted the discrete phase model (DPM) to simulate the air and dust flow around a solar PV panel based on the environment of Cairo. This research provides a qualitative comparison of the dust deposition on the solar PV panel due to different parameters such as the wind speed, dust particle size, and dust material. The results of the 2D simulations are summarized in the following points:

- Dust deposition rate increases by increasing the dust particle diameter for wind velocities above 2 m/s.
- For wind velocity of 2 m/s, dust deposition rate reaches its peak value at particle diameter of 150 μm, then starts to decrease.
- For small dust particles of 1 and 10 μm, the deposition rate is extremely low below 0.6% for all velocities and dust materials.
- Increasing wind velocity decreases the dust deposition rate.
- For high wind speeds above 8 m/s, the dust deposition rate of different particle sizes is nearly having the same low value.
- For wind velocities lower than or equal to 2 m/s, there is a critical particle size before which increasing density causes dust deposition rate to increase. After the critical particle size, increasing the density causes the dust deposition rate to decrease.
- For all wind velocities higher than 2 m/s, increasing the material density causes the dust deposition rate to increase for all dust sizes. Consequently, dolomite has the highest deposition rate, and gypsum has the lowest deposition rate for all dust particle sizes.
- The maximum percentage of deposition rate equals 10.8% and occurs at the dolomite dust material at a wind speed of 2 m/s and particle diameter of 150 μm. The minimum values of deposition rate occur at the high wind speeds of 9 and 11.5 m/s and particle diameter of 1 μm, where all dust materials have nearly the same value of dust deposition rate of about 0.2%.

Understanding the effect of different environmental parameters on the dust deposition can further be utilized in predicting the performance loss of the solar PV panel. Moreover, understanding the dust deposition behavior allows us to find possible solution strategies and cleaning methods. This has already been done in a recent article by the same present research team, Dagher and Kandil (2022). The article presents a novel passive cleaning method for the solar PV panels based on the environment of Cairo, Egypt.

## Data Availability

The datasets generated during and/or analyzed during the current study are available from the corresponding author on reasonable request.
